# Is Social Media Causing Anorexia? A Case Report on Kpop and Cultural Awareness

**DOI:** 10.1192/j.eurpsy.2023.1736

**Published:** 2023-07-19

**Authors:** J. Kim, K. Tran, P. Korenis, A. Sareen

**Affiliations:** Bronxcare Health System, New York, United States

## Abstract

**Introduction:**

One of the most significant changes to society came with the advent of social media, and with it a cultural shift in whom people consider their actual friends. The cultural influence of entertainment figures is not a new phenomenon; however, there has a revolution in the way celebrities interact with their fans, specifically in the Korean Pop (Kpop) industry. In contrast with musicians who release an album and then disappear into mysterious obscurity, Kpop stars constantly interact with fans through meet and greets, live streams, variety tv shows, and most importantly, through social media. With a concomitant rise in parasocial interactions and relationships, Kpop blurs the line between what constitutes pathological delusions and healthy fan activity.

**Objectives:**

- To learn the assessment and management of patients with anorexia nervosa
- To understand changes in management to address suicidality in patients with anorexia nervosa
- To understand influences by media in perpetrating certain body types in impressionable adolescents

**Methods:**

Patient is a 19 year-old Hispanic female with a Past Psychiatric History of Bipolar Disorder, who was brought in by EMS due for agitation and disorganized behavior. Patient presented manic, labile, and her delusions extended to beliefs that the Kpop group EXO has been communicating with her through morse code in their videos, and that certain members would wink at her through the computer screen in real time. The patient’s BMI at the time of admission was 15.4, and she continued to compare her own body to Kpop idols.

**Results:**

Patient shared a lifetime mix of both shame and trauma concerning her eating habits, with multiple incidents that may have contributed to her fear of eating, and simultaneously into her becoming obsessed with the Kpop group (“2018 and COVID were a miracle for me. I got closer to EXO”). Patient denies looking ugly or fat and seems mostly satisfied with her current appearance.

**Conclusions:**

With social isolation growing due to the pandemic, online parasocial relationships are becoming an increasingly normal part of people’s lives. We discuss a case where an unhealthy obsession with Kpop contributed to body dissatisfaction, and the precipitating factors that lead to these circumstances, as well as the challenges that are present in helping these adolescents and young adults in coping with social media use. As such, it is important to discuss the challenges faced by psychiatrists who must be sufficiently aware of the ever-changing face of contemporary cultural landscape when forming an accurate diagnosis.

**Disclosure of Interest:**

None Declared

